# The influence of previous robotic experience in the initial learning curve of laparoscopic radical prostatectomy

**DOI:** 10.1590/S1677-5538.IBJU.2016.0526

**Published:** 2017

**Authors:** José Anastácio Dias, Marcos F. Dall'oglio, João Roberto Colombo, Rafael F. Coelho, William Carlos Nahas

**Affiliations:** 1 Divisão de Urologia, Universidade de São Paulo Escola Médica, São Paulo, SP, Brasil

**Keywords:** Laparoscopy, Prostatectomy, Robotic Surgical Procedures

## Abstract

**Introduction:**

This study analyzed the impact of the experience with Robotic-Assisted Laparoscopic Prostatectomy (RALP) on the initial experience with Laparoscopic Radical Prostatectomy (LRP) by examining perioperative results and early outcomes of 110 patients. LRPs were performed by two ro-botic fellowship trained surgeons with daily practice in RALP.

**Patients and Methods:**

110 LRP were performed to treat aleatory selected patients. The patients were divided into 4 groups for prospective analyses. A transperitoneal approach that simulates the RALP technique was used.

**Results:**

The median operative time was 163 minutes (110-240), and this time significantly decreased through case 40, when the time plateaued (p=0.0007). The median blood loss was 250mL. No patients required blood transfusion. There were no life-threatening complications or deaths. Minor complications were uniformly distributed along the series (P=0.6401). The overall positive surgical margins (PSM) rate was 28.2% (20% in pT2 and 43.6% in pT3). PSM was in the prostate apex in 61.3% of cases. At the 12-month follow-up, 88% of men were continent (0-1 pad).

**Conclusions:**

The present study shows that there are multiple learning curves for LRP. The shallowest learning curve was seen for the operative time. Surgeons transitioning between the RALP and LRP techniques were considered competent based on the low perioperative complication rate, absence of major complications, and lack of blood transfusions. This study shows that a learning curve still exists and that there are factors that must be considered by surgeons transitioning between the two techniques.

## INTRODUCTION

Laparoscopic Radical Prostatectomy (LRP) was first described by Schuessler in 1992, and the first large series was published by Guilloneau (1, 2). As LRP becomes more established with more long-term follow-up studies available, this approach is showing solid oncological and functional results. The major barriers to the adoption of LRP are the technically challenging nature of the procedure and a steep learning curve (3).

The challenges presented by skill acquisition were overcome in part by the introduction of the da Vinci Surgical System that facilitates robotic-assisted laparoscopic prostatectomy (RALP). In Europe and the United States, RALP is now displacing radical retropubic prostatectomy as the gold standard surgical approach to treat localized prostate cancer, such that RALP may eventually completely replace LRP (4). However, RALP does have limited availability, training facilities and higher direct costs, which is an area of concern given the economic considerations that are becoming increasingly important for reasonable health care resource allocation in light of budgetary constraints and limited resources, especially in developing countries (5).

Surgeons who have experience with LRP could obtain excellent operative outcomes with RALP, accelerate procedural uptake and eliminate the RALP learning curve because of the similarities between the techniques (6). Moreover, given that RALP replicates the laparoscopic technique, experience with RALP should allow surgeons to perform LRP without a learning curve. This situation can occur in developing countries and in regions without access to robotic facilities. However, whether proficiency with one surgical technique ensures proficiency in the other is unclear (7, 8).

This study analyzed the impact of the experience with RALP on the initial experience with LRP by examining perioperative results and early oncological and functional outcomes of 110 prostate cancer cases.

## MATERIALS AND METHODS

Between November 2010 and August 2012, 110 LRP were performed by two surgeons to treat aleatory selected patients with clinically localized prostate cancer referred to the Instituto do Câncer do Estado de São Paulo (Cancer Institute of the State of São Paulo). Both surgeons participated in LRP during the residency with the same surgeon and were experienced in upper tract laparoscopic surgeries, coordinating together the oncologic laparoscopic program, mentoring urology residents and performing themselves at least 2-3 challenge surgeries weekly (partial laparoscopic nephrectomy, challenge radical laparoscopic nephrectomy) (9). The surgeons also performed post residency two years of fellowship training in RALP in the United States. At the time of the study, both were RALP proctors, mentoring robotic surgeries in another institution, and used to perform themselves 2-4 RALP weekly for the past three years before the beginning of the study. Nevertheless, it was not possible to evaluate the previous surgical experience of both surgeons because they used to perform in many institutions.

The patients were operated by the surgeons alternately and ordered chronologically. Data were collected prospectively and all the patients were divided into 4 groups of approximated size for analyses. A transperitoneal approach that simulates the RALP technique, as described by Patel et al. (10), was used for all patients, and neuro-vascular bundle preservation was attempted in clinically localized cancers. All complications were graded according to the Clavien-Dindo classification. Prostatic Specific Antigen (PSA) tests were performed after 6 and 12 months. Biochemical recurrence was defined as PSA >0.2μg/L or PSA that never fell below 0.1μg/L. Continence was defined as 0 or 1 (confidence) pad per 24h. All specimens were reviewed by a specialist uropathologist. Positive surgical margins (PSM) were defined as the presence of tumor at the inked surface of the specimen (11).

Statistical analysis was performed with the IBM^®^ SPSS^®^ Statistics 23 program. Statistical analyses were carried out using Fisher's exact test and non-parametric Kruskal-Wallis test for qualitative variables. ANOVA was used to compare continuous values and the Tukey test was applied to explore differences between groups. Logistic regression curves were used to represent the tendencies relative to experience.

## RESULTS

Statistical analyses found no statistical difference in age, body mass index, PSA, clinical stage, pathologic staging, and biopsy Gleason score between each phase of the learning curve (Table-1).

**Table 1:** Demographic and Clinical Characteristics.

Characteristic		Group					p value
		Total	1	2	3	4	
**Age (years)**							
	Mean	61.7	61.7	63.7	61.2	60.2	0.244^1^
	SD	6.30	8.18	5.50	4.91	6.24	
**Body Mass Index**							
	Mean	25.97	25.49	27.10	25.84	25.35	0.180^1^
	SD	3.22	2.96	2.70	4.12	2.992	
**PSA (μg)**							
	Median	7.5	7.5	8.3	9.3	5.8	0.072^2^
	Range	6.5-63.53	6.9-23.5	6.72-62.4	7.22-16.7	3.26-16.2	
**Clinical Stage, (%)**							
	cT1	64 (58.2)	18 (66.7)	11 (40.7)	17 (63.0)	18 (64.3)	
	cT2	45 (40.9)	9 (33.3)	16 (59.3)	10 (37.0)	10 (35.7)	0.579^3^
**Biopse Gleason Score n(%)**							
	<7	77 (70.0)	21 (77.8)	17 (63.0)	18 (66.7)	21 (75.0)	
	7	26 (23.7)	6 (22.2)	7 (25.9)	7 (25.9)	6 (21.4)	0.246^3^
	>7	6 (5.5)	0 (0.0)	3 (11.1)	2 (7.4)	1 (3.6)	

Note:

1 ANOVA

2 Kruskal-Wallis

3 Fisher.

**Figure 1:**
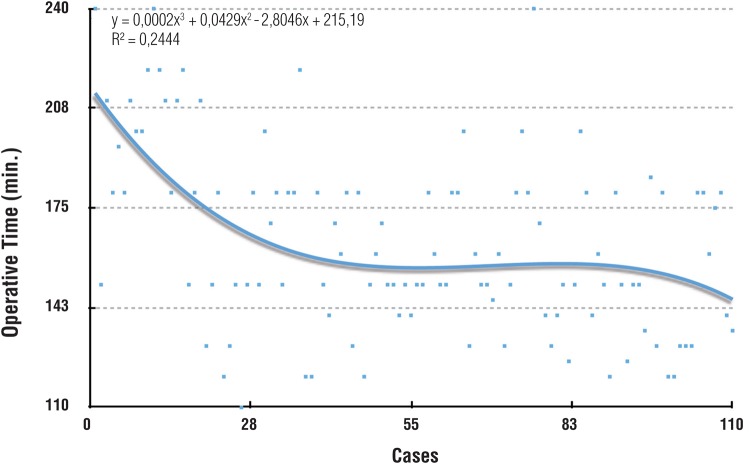
Learning curve for operative time.

The median operative time was 163 minutes (range 110-240), with a significant reduction along the experience (p=0.0007) (Table-2). After a significant decrease until case 40, a plateau was reached. After case 90, a new reduction appeared (Figure-1).

**Table 2:** Perioperative Data.

Characteristic		Group					
		Total	1	2	3	4	p
**Operative Time (min)**							
	Mean	163.54	182.41	160.37	160.93	151.38	0.0007^1^
	SD	29.97	36.88	24.41	25.27	23.98	
**Blood Loss (mL)**							
	Median	250	200	250	250	300	0.6393^2^
	Range	250-950	250-900	250-900	300-750	275-800	
**Hospital Stay (days)**							
	Median	1	1	1	1	1	0.0593^2^
	Range	0-13	1-2	0-2	0-2	0-13	
**Continence (%)**		88.0	74.1	92.3	88.9	96.4	0.1012^3^
**Complication (%)**							
	Clavien grade I / II	8 (7.3)	3 (11.1)	2 (7.4)	1 (3.6)	1 (7.2)	0.5619^3^
	Clavien grade III	8 (7.3)	2 (7.4)	0 (0.0)	4 (14.3)	2 (7.1)	0.2644^3^
**T Stage (%)**							
	T2	70 (63.6%)	18 (69.2%)	19 (70.4%)	18 (66.7%)	15 (51.7%)	0.2265^3^
	T3	39 (35.4%)	8 (30.8%)	8 (29.6%)	9 (33.3%)	14 (48.3%)	

Note:

1 ANOVA

2 Kruskal-Wallis

3 Fisher.

The median blood loss during surgery was 250mL (range 50-1000mL), and there was no statistical difference in the series (P=0.6393) (Table-2). No patients required a blood transfusion. Complications were uniformly distributed along the series (P=0.6401) (Table-2). Rectal lesions occurred in 2 patients (1.81%), and were repaired intra-operatively. Conversion was necessary in 1 (0.90%) patient due to fibrosis after biopsy. There were two incisional hernias at the vertical infra umbilical port that required surgery during the first year of follow-up. In addition, there was one clinical anastomotic leak that required bilateral ureteral stents, one anastomotic stricture that required internal urethrotomy, and one patient required cystoscopy to reposition the urethral catheter on the first post-operative day. There were no life-threatening complications or deaths (Clavien IV and V) in the series.

**Figure 2:**
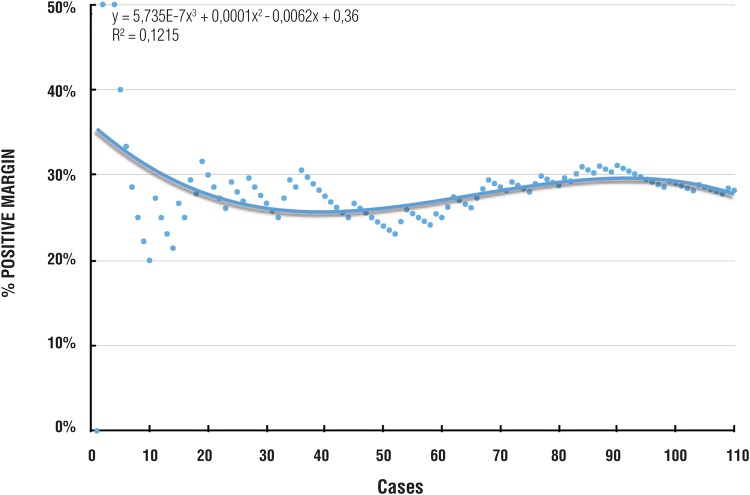
Positive surgical margin rate.

The overall PSM rate was 28.2%, corresponding to 20% in pT2 and 43.6% in pT3. There was no clear decreasing tendency and the PSM was persistently between 25 and 30% (Figure-2). A comparison of the pT2 (p=0.3818), pT3 (p=0.7993) and overall (P=0.6661) groups showed no statistical difference. PSM was in the prostate apex in 61.3% of cases and the location showed no difference with experience (p=0.7533).

**Figure 3:**
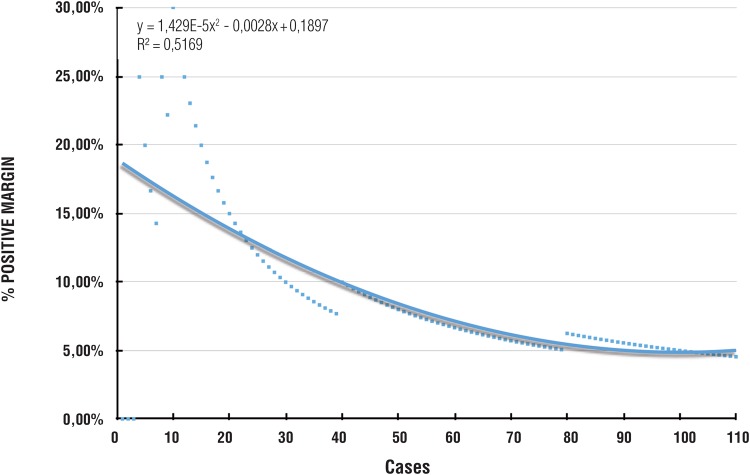
Incontinence rate.

At the 12-month follow-up, 88% of men were continent. The continence rate tended to decrease up to case 70 when it reached a plateau of ~95% continence rate (Figure-3).

## DISCUSSION

The feasibility and reproducibility of LRP have been established and long-term oncological and functional outcomes have been shown to be comparable with open retropubic radical prostatectomy. LRP offers advantages over open radical prostatectomy in terms of decreased blood loss, analgesic requirements, hospitalization and convalescence periods (12). However, the steep learning curve is a hurdle to wide uptake of this surgical approach. Previous studies showed that fellowship training significantly reduces the learning curve without compromising safety or outcomes (7, 13–15).

A limited number of mentorship programs in LRP currently exist in developing countries, and most surgeons who perform LRP were trained in the United States or Europe, where RALP now accounts for 95% of minimally invasive prostate cancer treatment (6). Due to budget limitations, this transition to a robotic surgical interface will be much more gradual in developing countries (9, 16).

Although the open radical prostatectomy would be a more realistic option considering the cost saving (17), LRP remains a cost-effective minimally invasive surgical option especially when the operative time is shorter than 4 hours and the use of reusable instruments are optimized (5).

Many studies have assessed the learning curve of RALP for surgeons transitioning from LRP to the robotic technique. These studies showed that technical similarities between LRP and RALP can help minimize the learning curve, particularly when surgeons are already proficient in antegrate prostate dissection techniques and laparoscopic principles (18). It was suggested that the skill set in other laparoscopic procedures, such as radical and partial nephrectomy is transferable to LRP (16). Hence, the learning curve for LRP would likely be lessened for laparoscopically experienced and RALP-trained surgeons, which in turn translates into improved initial outcomes for patients.

This study analyzed the early results of the first 110LRP performed by two surgeons experienced in RALP and upper tract laparoscopy at a university teaching hospital to describe the challenges that surgeons trained in RALP face when they try to initiate practice in areas without the robotic system.

The mean operative time was 163.5 minutes, with no cases requiring more than 240 minutes, and there was only one conversion. After 40 cases, the learning curve plateaued at 150 minutes, which is comparable to results from large series studies. Even recognizing that the operative time is not as relevant to the patient, the value could represent technical difficulties or indicate the lack of progression in any step of the surgery (3). A similar abrupt reduction in the operative time after only a few cases was described by several studies, suggesting that 15 to 25 cases is sufficient to achieve a mean operative time of 3-4 hours, and could represent the adaptation to laparoscopic instruments and maneuvers, suggesting that the principal steps were already learned (16, 19). The amount of blood loss and length of hospital stay were stable and comparable to large series. There were no cases with high blood loss volumes and none of the patients required transfusions (14, 20).

The complication rate was 14.6%, with half being Clavien I/II and half being Clavien III. No Clavien IV or V complications were seen. There was only one open conversion due to bleeding after prostate removal that may have been caused by post-biopsy fibrosis. Mitre et al. found a significant reduction in the complication rate, mainly limited to transfusions and urinary extravasation (9). Hruza et al. analyzed the complications in 2.200LRP cases and described complication rates of 21.7% (Clavien 1 and 2) and 11.5% (Clavien 3-5), as well as a significant reduction in minor complications when comparing the first and last 200 cases (11). Siqueira et al. warned about the possibility of major complications to occur during the learning curve and found no difference between the trans and extra peritoneal approach (21) The lack of a significant reduction in complications may have been due to the low complication rate since the outset of our study, and favors the hypothesis that expertise transfers between the techniques.

LRP is considered to be a well-established procedure with proven benefits in terms of reduced preoperative bleeding and need for transfusion (20). After 1138 cases, Soares et al. found a median bleeding of 200mL (10-1.300mL) that stabilized after 150 cases and a transfusion rate of 0.5%, which is similar to that seen by Stolzenburg et al. among 2.000 cases (13, 22). Moreover, our initial results were comparable to those described in a study by Good et al., which showed reduced bleeding after 500 cases (23).

Since the main goal of LRP is oncologic success, initial experience could be based on a PSM rate that should be 0%, but in practice 15% is considered acceptable. Our PSM rates were 20% in pT2 and 43.6% in pT3 with no statistical improvement with experience, were comparable to many previous reports (2, 3, 11, 12, 20, 24), although it was higher than series with PSM rates between 7.2 and 13.9%, probable due to the higher proportion of pT3 in our series (22, 25). Many studies also described a plateau in the PSM after 250 cases (3, 7, 23), while for others the plateau occurred after 100 cases (26). All of these studies defended the need for a continuous evaluation of outcomes, modulated teaching methods, and revision of video recordings to provide better outcomes and minimize the learning curve. However, more experience-between 500 and 1.000 cases-may be needed to achieve a PSM plateau for pT3 tumors, which may have the steepest learning curve (23, 27).

The high PSM rates in our series were associated with a high incidence of apical margin that was present in 61.3% of PSM. This outcome could be due to the limited number of cases that was not sufficient to overcome the initial learning curve, to the attempts to preserve the neuro-vascular bundles in clinically under staged patients and to technical difficulties associated with attempts to reproduce the robotic technique in the apex dissection in the absence of the freedom afforded by articulating instruments (6).

McNeill et al. suggested that frozen sections be routinely used to reduce the apical margins that accounted for 53% of their overall PSM (26). Meanwhile, Good et al. compared the learning curves and outcomes for LRP and RALP and found that RALP yielded significant benefits to patients compared to LRP, especially outcomes that were linked to better apical dissection (apical PSM and continence), and considered that this improvement may be related to the technological platform rather than factors associated with individual surgeons (23).

For continence, we had concern that our patients would find the international validated questionnaire to be too complicated. Moreover, different definitions of continence may contribute to a difference of about 10% in continence rates. As such, we chose to use the simplified criteria of ‘no drops, no pad’, that, in practice, includes the patients who uses one pad a day for his reassurance as well as the patient who leaks a few drops but does not uses a pad. In this study, the continence rate plateaued at 70 cases wherein 95% of patients were continent. This result supports the thinking that previous experience with RALP may facilitate competence with LRP. A study by McNeill et al. described similarly a plateau after 250 cases following modular training. Similar overall continence rates have been described, however more cases are required to reach overall continence rates that exceed 95% (22, 26).

Our study has several limitations. The non - randomized nature, relatively few patients and the short follow-up that limits the usage of biochemical recurrence in our study. Erectile dysfunction was not evaluated in our series due to no application of validated questionnaires. No quality of life measures were recorded to investigate patient perceptions of their outcome. It should be considered that any previous radical prostatectomy experience has potential to improve the results independent of the surgical technique utilized.

In addition, this data relates to two specific surgeons and the results may not necessarily extrapolate to other centers. This limits the applicability of our comments to all surgeons transitioning between the techniques of radical prostatectomy, as differing levels of aptitude and prior exposure will heavily impact on the results. The informative power of an institutional learning curve might be limited, because it is difficult to determine if the surgeons were equally skilled or if one struggle versus the others (11).

## CONCLUSIONS

The present study shows that there are multiple learning curves for LRP, and support the idea that self-evaluation and continuous monitoring of surgical outcomes are needed to develop interventions that will improve surgeon performance. The shallowest learning curve was seen for the operative time. The PSM learning curve may need additional experience to improve the results, principally in the apical margin. Surgeons transitioning between the RALP and LRP techniques were considered competent based on the low perioperative complication rate, absence of major complications, and lack of blood transfusions. This study provides an overview of early LRP results for two surgeons trained in RALP and shows that a learning curve still exists and that there are factors that must be considered by surgeons transitioning between the two techniques.

## ABBREVIATIONS

LRP: = Laparoscopic Radical Prostatectomy
RALP: = Robotic-Assisted Laparoscopic Prostatectomy
PSA: = Prostatic Specific Antigen
PSM: = Positive Surgical Margins
